# Spinoglenoid Notch Ganglion Cyst: A Case Report

**DOI:** 10.7759/cureus.39279

**Published:** 2023-05-20

**Authors:** Mohammed Alsabieh, Mosa Alzahrani, Abdulaziz Almuhanna, Najla Bedaiwy

**Affiliations:** 1 Orthopedic Surgery, Prince Mohammed Bin Abdulaziz Hospital, Riyadh, SAU; 2 College of Medicine, King Saud Bin Abdulaziz University for Health Sciences, Riyadh, SAU

**Keywords:** nsaids, suprascapular nerve neuropathy, magnetic resonance imaging, decompression, spinoglenoid notch ganglion cyst

## Abstract

Suprascapular nerve dysfunction caused by ganglion cysts is a rare condition that can cause significant pain and weakness in the shoulder. The suprascapular nerve is a branch of the brachial plexus that innervates the supraspinatus and infraspinatus muscles. It is most commonly compressed at the suprascapular notch or the spinoglenoid notch. A 40-year-old male presented with a two-year history of left shoulder pain that was aggravated by overhead activities. Physical examination revealed mild tenderness along the infraspinatus with noticeable atrophy, full range of motion, and mild external rotation weakness. MRI was obtained and confirmed the suspected diagnosis of suprascapular nerve dysfunction caused by a ganglion cyst at the spinoglenoid notch. The patient was initially treated conservatively with physical therapy and non-steroidal anti-inflammatory drugs (NSAIDs), but after completing nine months of conservative management, he showed no improvement. The patient elected to undergo open ganglion cyst excision and decompression of the spinoglenoid notch. Postoperatively, the patient's pain resolved gradually and he regained the full power of external rotation. The patient was followed for one year postoperatively and was satisfied with the outcome with a full range of motion, full power, and a complete return to his baseline level of activity. In conclusion, this case report demonstrates the successful treatment of suprascapular nerve dysfunction caused by a ganglion cyst at the spinoglenoid notch with open ganglion cyst excision and decompression. This procedure is a safe and effective treatment option for patients with this condition who have failed to respond to conservative treatment and emphasizes and signifies the role of eliciting a detailed patient history, conducting a thorough radiographic examination including MRI scans, and planning optimum surgical interventions

## Introduction

Ganglion cyst-inducing suprascapular nerve dysfunction is a rare presentation that can cause severe shoulder pain and weakness. The supraspinatus and infraspinatus muscles are innervated by a brachial plexus branch known as the suprascapular nerve. The suprascapular or spinoglenoid notches are the most common sites of compression. The suprascapular notch is a small opening in the scapula through which the suprascapular nerve passes. The spinoglenoid notch is a small depression on the posterior surface of the scapula where the suprascapular nerve passes through to innervate the infraspinatus muscle. Compression of the suprascapular nerve can be caused by several factors, including trauma, overuse, and space-occupying lesions such as tumors, ganglion cysts, and paralabral cysts.

The clinical presentation of suprascapular nerve dysfunction can vary depending on the site of compression. Patients may complain of shoulder pain, weakness in abduction and external rotation, and atrophy of the supraspinatus and infraspinatus muscles. The diagnosis of suprascapular nerve dysfunction is made based on the patient's history and physical examination. Imaging studies such as MRI can be helpful in confirming the diagnosis and identifying the site of compression.

Treatment of suprascapular nerve dysfunction depends on the cause of the compression. In some cases, non-surgical treatment such as physical therapy and injections may be effective. In other cases, surgery may be necessary to decompress the nerve. This report is of a patient with suprascapular nerve dysfunction caused by a ganglion cyst at the spinoglenoid notch.

## Case presentation

A 40-year-old male medically free, presented to our clinic complaining of vague left shoulder pain that started two years prior to his presentation. There was no history of trauma to the shoulder or any other initial event. His pain was tolerable at first, but it worsened over time, the pain was aggravated by activities of daily living (overhead activities in particular) and relieved by simple analgesia (paracetamol and non-steroidal anti-inflammatory drugs (NSAIDs)). The patient sought medical advice in another hospital and was diagnosed to have a ganglion cyst compressing the suprascapular nerve at the glenoid notch. He underwent arthroscopic decompression but unfortunately, there was no relief of the symptoms.

**Figure 1:**
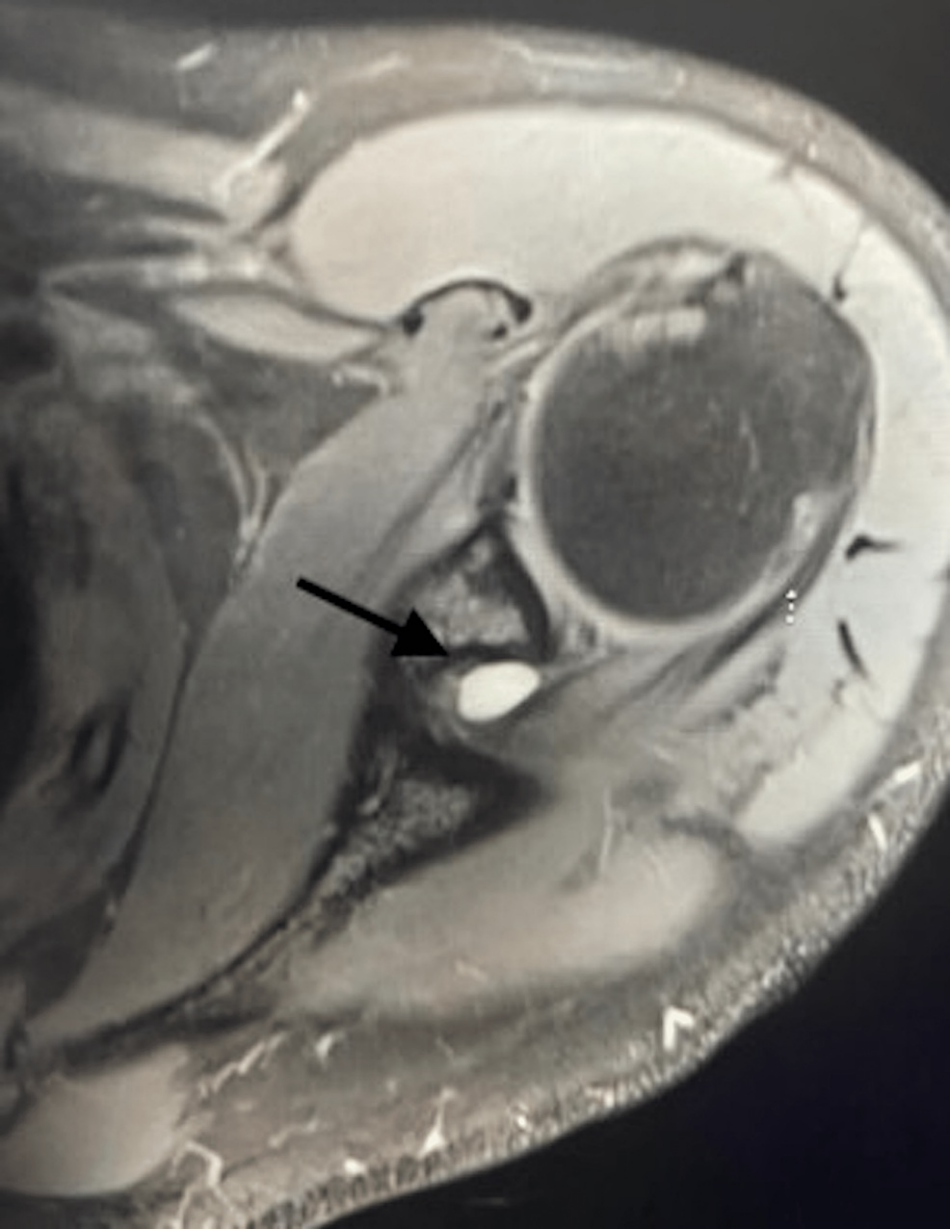
Axial MRI scan of the left shoulder showing the spinoglenoid notch ganglion cyst

**Figure 2:**
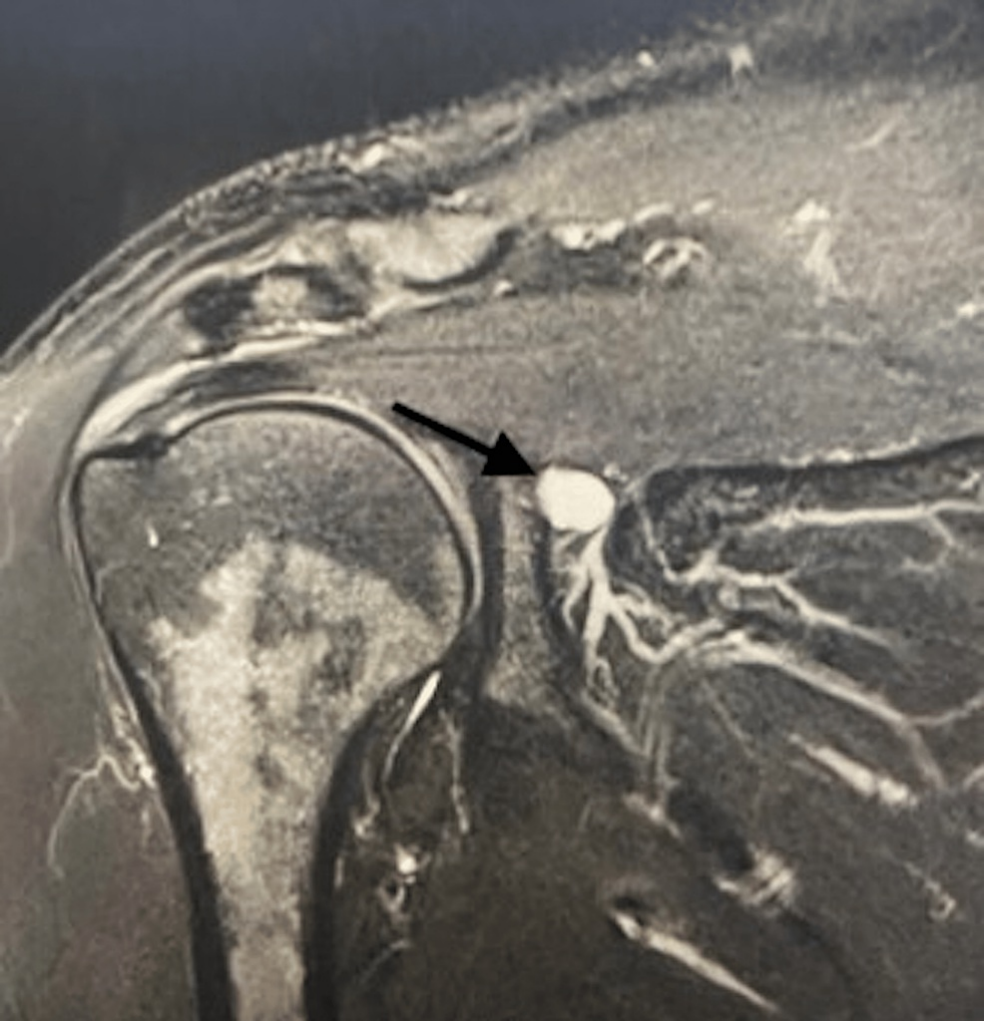
Coronal MRI scan of the left shoulder showing the spinoglenoid notch ganglion cyst

**Figure 3:**
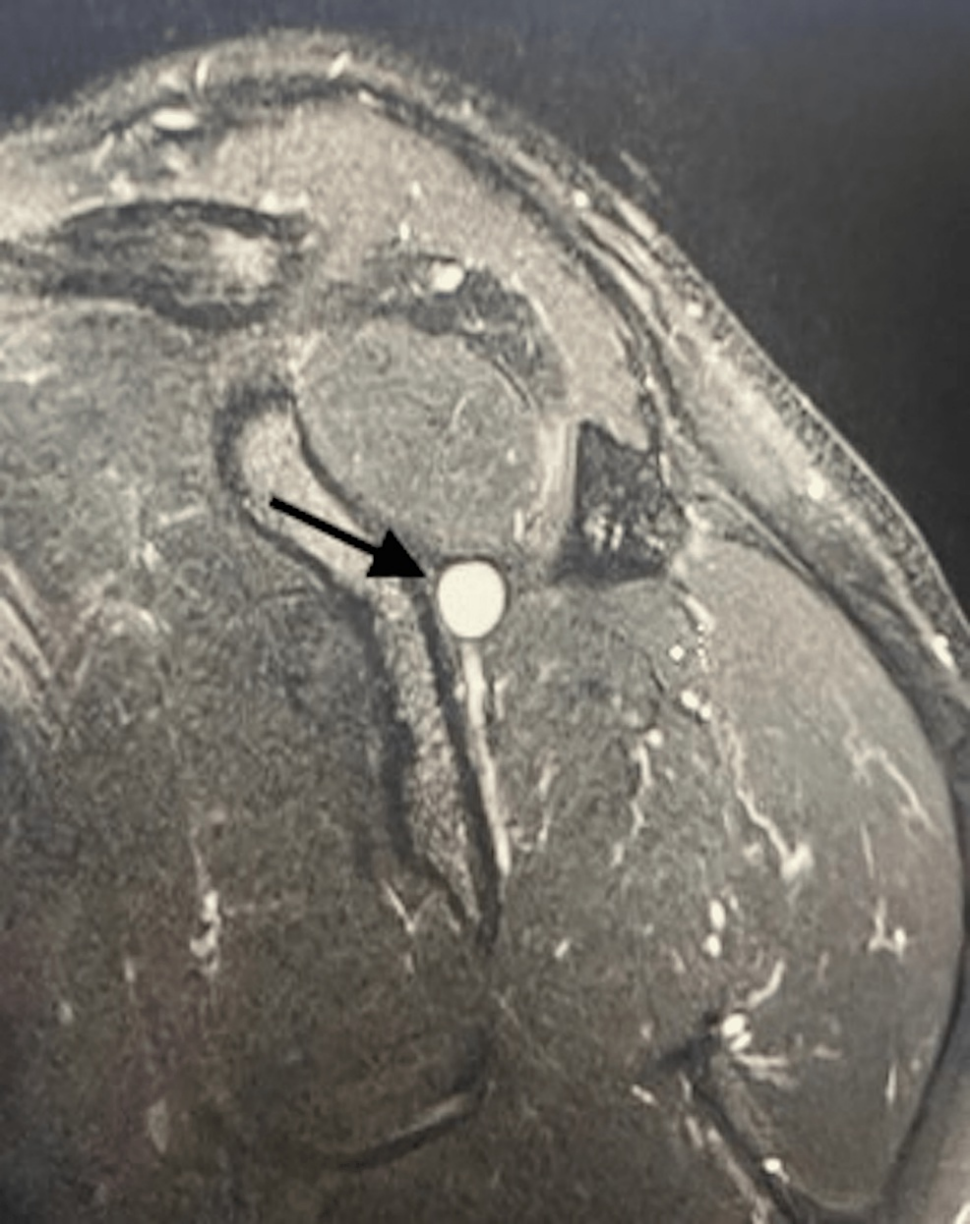
Sagittal MRI scan of the left shoulder showing the spinoglenoid notch ganglion cyst

Physical examination revealed mild tenderness along the infraspinatus with noticeable atrophy, full range of motion, and mild external rotation weakness. MRI was obtained and confirmed the suspected diagnosis (Figures 1-3). The patient was initially treated conservatively with physical therapy and NSAIDs, but after completing nine months of conservative management, he showed no improvement. The patient elected for open ganglion cyst excision and decompression of the spinoglenoid notch. 

Surgical technique

The patient was in prone position, under general anesthesia. A longitudinal incision was made 3cm from the posterolateral edge of the acromion toward the posterior axillary fold, the deltoid was split along its fibers, and the infraspinatus fascia was incised and reflected off the scapular spine. The spinoglenoid notch was visualized and the spinoglenoid ligament was released; the nerve was seen free of compression, protected at all times. The cyst was resected and sent to histopathology. Histopathology showed the typical histological appearance of a ganglion cyst.

Postoperatively, the pain resolved gradually, and the patient regained full power of external rotation. The patient was followed for one year postoperatively and was satisfied with the outcome with a full range of motion, full power, and a complete return to his baseline level of activity.

## Discussion

The suprascapular nerve originates from the upper trunk of the brachial plexus and receives innervation from brachial plexus C5 and C6. It courses down posterior to the omohyoid muscle before it enters the suprascapular notch, which is boarded superiorly by the transverse scapular ligament. It gives motor branches to the supraspinatus within 1cm after exiting the notch. Then it descends inferiorly and obliquely along the inferior surface of the supraspinatus to enter the spinoglenoid notch and then it gives motor branches to the infraspinatus [1,2].

The suprascapular nerve is compressed usually at two common sites: the suprascapular notch and the spinoglenoid notch with almost comparable rates of occurrence. The clinical presentation can vary depending on the site of compression. Suprascapular nerve compression at the suprascapular notch was first described in 1959 [3], and at the spinoglenoid notch in 1989 [4].

Suprascapular nerve pathology can be caused by a variety of etiologies, one of which is trauma, which has been described as one of the frequent causes of dysfunction including scapular fractures, proximal humerus, and shoulder dislocation [3,5]. Moreover, overuse activities were also described as one of the mechanisms of suprascapular nerve dysfunction [6]. Compression can be caused by space-occupying lesions such as tumors, ganglion cysts, paralabral cysts, and hematomas [7]. 

Diagnosing suprascapular nerve dysfunction can be very challenging as it mimics the clinical presentation of those of more common conditions, for example, tumors, rotator cuff tear, labral tear, impingement syndrome, and adhesive capsulitis. Patients typically complain of chronic poorly localized shoulder pain of mostly the posterolateral part of the shoulder. Possible radiation to the neck, arm, or upper chest was reported as well. The pain can be aggravated by positions that increase the tension over the nerve or the ligaments, it is usually worse with overhead activities and adduction with internal rotation. Patients may give a history of direct trauma or activities that require repetitive use of the shoulder [8].

Careful physical examination is often very helpful in raising the index of suspicion of suprascapular nerve dysfunction. Positive findings include abduction and external rotation weakness, wasting of the infraspinatus and supraspinatus (if the compression is at the suprascapular notch), and tender suprascapular and spinoglenoid notch, although the localization of these areas can be extremely difficult [8].

Electromyography (EMG) and nerve conduction study (NCS) remain the gold standard for diagnosing suprascapular nerve dysfunction. These studies can be used to diagnose and assess the extent of nerve dysfunction and associated pathologies. EMG and NCS measure the electrical activity of muscles and nerves, while MRI creates detailed images of the body. These tests can be used together to provide a more complete picture of the condition and help healthcare providers make a diagnosis and develop a treatment plan and help confirm the diagnosis and locate the site of compression. Findings include motor loss in the infraspinatus or both the supraspinatus and infraspinatus muscles, depending on the level of the lesion, denervation potentials, and prolonged motor latencies. The sensitivity and specificity of NCS and EMG can be up to 91% as reported by various studies [9]. In addition, MRI must be included in the workup of any suspected soft tissue pathology around the shoulder. It can be extremely valuable in evaluating compressive lesions, and assessing the extent and severity of the compression according to the degree of muscle atrophy. Also, it can detect concomitant pathologies such as rotator cuff and labral tears [10].

Treatment of suprascapular nerve dysfunction depends on a number of factors: the presence or absence of space-occupying lesion, the site of dysfunction, and the response to non-surgical modalities. In the absence of space-occupying lesions, there is almost a consensus on managing the patient conservatively. Conservative management includes rest physiotherapy and NSAIDs. Physical therapy can help in maintaining the shoulder range of motion and strengthening rotator cuff muscles [11,12]. If nonoperative modalities fail to alleviate the symptoms, multiple minimally invasive options can be utilized. Suprascapular nerve injections with local anesthetic agents and steroids are a good illustration of minimally invasive modality [13], Other options are neurostimulation [14], cryoneurolysis [15], and pulsed radiofrequency ablation [16].

Surgical management is considered in cases of failure of conservative management and the presence of space-occupying lesions. Surgical release of the spinoglenoid ligament has been proven to improve the symptoms of pain and weakness in a number of cases; however, there are no studies comparing the outcome of conservative versus surgical release of the spinoglenoid ligament. Therefore, it is recommended to treat entrapment of the nerve due to ligamentous impingement conservatively for at least six months before attempting surgical release [17]. Compression of the nerve at the spinoglenoid notch due to a lesion, especially ganglion cysts, responds very poorly to non-surgical management, despite the chances of spontaneous resolution. 

Surgical options for compression by ganglion cyst at the spinoglenoid notch include radiologically-guided aspiration, open excision, and arthroscopic decompression with labral and rotator cuff tear, or a combination of the aforementioned modalities. Significant improvement in symptoms following surgical decompression has been reported in numerous studies [7,8]. Cyst aspiration showed recurrence rates of 48% after following the patients for two years and a primary failure of rate 18% [18]. Comparing open versus arthroscopic decompression, arthroscopic decompression showed lower rates of recurrence with better access to address concomitant labral tears and similar outcomes in regard to symptom relief [7,18].

Our patient had disabling shoulder pain involving mainly the posterolateral aspect for more than two years, with significant weakness of both abduction and external rotation and noticeable atrophy of the infraspinatus. The diagnosis was confirmed by MRI, which showed a cyst compressing the nerve at the spinoglenoid notch, and a labral tear was excluded. The patient underwent arthroscopic decompression prior to his presentation to our hospital with failure to improve the symptoms. The patient elected to undergo open excision, which was done and provided significant symptomatic relief with no recurrence at the time of the last follow-up, one year after the surgery.

## Conclusions

This report is among the few of this rare condition, reported from Saudi Arabia. It highlights the challenges in diagnosing suprascapular nerve dysfunction, as it can mimic the clinical presentation of other more common conditions. Careful physical examination and imaging studies, such as MRI, are essential for making an accurate diagnosis. Early diagnosis and treatment of suprascapular nerve dysfunction are important to prevent permanent muscle atrophy and weakness. Non-surgical treatment such as physical therapy and injections may be effective in some cases and surgery may be necessary to decompress the nerve in other cases. The overall treatment plan needs to adequately address the patients’ functional disturbances and should mitigate the risk of potential neuro-vascular involvement.
